# Orthodontic patients and the information found on the web: a cross-sectional study

**DOI:** 10.1186/s12903-023-03609-4

**Published:** 2023-11-13

**Authors:** Roberta Crispino, Alice Mannocci, Irene Alfonsa Dilena, Jackson Sides, Federico Forchini, Wael Mohammad Asif Alherawi, Sylvia A. Frazier-Bowers, Cristina Grippaudo

**Affiliations:** 1 Department of Biomedical, Surgical and Dental Sciences, Ospedale Maggiore Policlinico, University of Milan “La Statale”, Fondazione IRCCS Cà Granda, Milano, Italy; 2 Faculty of Economics, Universitas Mercatorum, Rome, Italy; 3 Department of Orthodontics, University of Ferrara, Ferrara, Italy; 4 University of North Carolina Adams School of Dentistry, Chapel Hill, NC USA; 5 Finnish National Dental Care, Oulu, Finland; 6 Private practice, Madinah, KSA Saudi Arabia; 7 Orthodontics and Oral Facial Genetics Associate Dean, Student Affairs and Admissions Chief Diversity Officer, Indiana University School of Dentistry, Indianapolis, USA; 8 Dental Institute, DDS, Catholic University of the Sacred Heart, Catholic University of the Sacred Heart Fondazione Policlinico Gemelli IRCCS, Milano, Italy

**Keywords:** Decision-making, Digital information, Digital natives, e-health, Generational differences, Orthodontics, Survey

## Abstract

**Background:**

In recent years, the Web has become a source of medical information for patients, even though the information available online may be incorrect or qualitatively inadequate. Younger generations, immersed in a digital environment since a very tender age, are more likely to get informed online. This study aims to understand the relevance of online information for prospective orthodontic patients and to investigate the effects of digital research on patients’ decision-making process, and it also aims to investigate potential generational differences between digital natives and digital immigrants.

**Materials and methods:**

An anonymous questionnaire was developed to investigate patients’ orthodontic-themed Web searches as well as the effects digital material had on their decision-making process. Before submitting the newly designed survey to patients, it was validated in a pilot study. Univariate analysis was applied to analyze the relationship between the demographic characteristics of respondents and their answers on the use of digital research for the decision-making process.

**Results:**

64.6% of the study population searched the Web for orthodontic information prior to their visit. Google was the most used platform regardless of patients’ age. The perceived reliability of online sources varied significantly with age. Men displayed more trusting behavior towards their doctor than women. Prospective patients’ satisfaction with affected patients’ decision-making processes, and the perceived reliability of online sources of information had repercussions on the doctor-patient relationship.

**Conclusions:**

Orthodontists should be aware that the majority of patients use the Internet as a source for orthodontic information, and that patients who are digital immigrants are more prone to trust the information found online. Patients who perceive the information found on the Web as either useful or reliable don’t easily discard it, even if it is inconsistent with the orthodontist’s opinion.

## Background

Technological advancements, (e.g. the continually evolving Internet, social networks, and smartphones) have dramatically changed lifestyles and habits of consumers. In 2022 consumers who accessed the Web were estimated to be 5.5 billion, 69% of the World’s total population [1]. The digital divide has decreased with more accessible and user-friendly tech tools replacing traditional communication media. As a result vast amounts of information ranging from lifestyle, entertainment, and healthcare advances are one click away.

Furthermore, the tendency to deploy tech and digital platforms (Tik Tok, Instagram; YouTube, etc.) has rapidly increased in the millennial and Gen Z generations. In 2001 Marc Prensky, a writer from the U.S., coined the phrase “digital natives” to indicate the people who grew up surrounded by digital technologies [2]; this group includes all people born since 1985, individuals born before are identified as “digital immigrants” instead. Teenagers and young adults tend to use technology more frequently and with more ease than more mature individuals.

Over the years, the number of patients who search the Web and social networks for information concerning medical conditions or therapies has increased. This is true in the orthodontic field as well [3]. However, not all the information available on the Web is clinically accurate [4–6] or of adequate quality [7–10]. As a possible consequence, patients might ask for treatments that are neither state-of-the-art nor based on scientific evidence, eliminating the orthodontist’s clinical experience and knowledge. Given the immersion into a nearly exclusive digital space, younger generations are likely to seek medical information online more frequently than digital immigrants.

This study aims to understand the impact of online information for prospective and current orthodontic patients, and to investigate the effects of digital research on patients’ decision-making process. It also aims to compare digital natives and digital immigrants to uncover generational differences in obtaining and processing orthodontic information.

## Materials and methods

### Study design and participants

This was a cross-sectional study using a self‐administered questionnaire, conducted from October 2020 to January 2022. The questionnaire was distributed in the following facilities: Policlinico Universitario Agostino Gemelli – Rome (Italy), University of Oulu (Finland), University of North Carolina (USA), and Dr. W. A.’s private practice (Saudi Arabia).

The eligibility criteria for participation in the study were:

- Adult orthodontic patients or parents/legal representatives of underage orthodontic patients;
- Respondents aged ≥ 18 years old;
- Ongoing or planned orthodontic treatment.

Respondents were divided into two groups according to age:

- Digital natives (DN): respondents born in 1985 or later;
- Digital immigrants (DI): respondents born before 1985.

The study protocol was approved by the Ethical Committee of Fondazione Policlinico Universitario A. Gemelli, Rome (protocol ID: 2922). The results are presented following the STROBE guidelines [11].

### Survey design

To investigate orthodontic patients’ Internet use and its perceived reliability, an anonymous questionnaire was developed (Fig. 1a and b -2). The carefully constructed questionnaire assessed how respondents gathered orthodontic information, and the contribution of digital material to their decision-making process.

**Fig. 1a:**
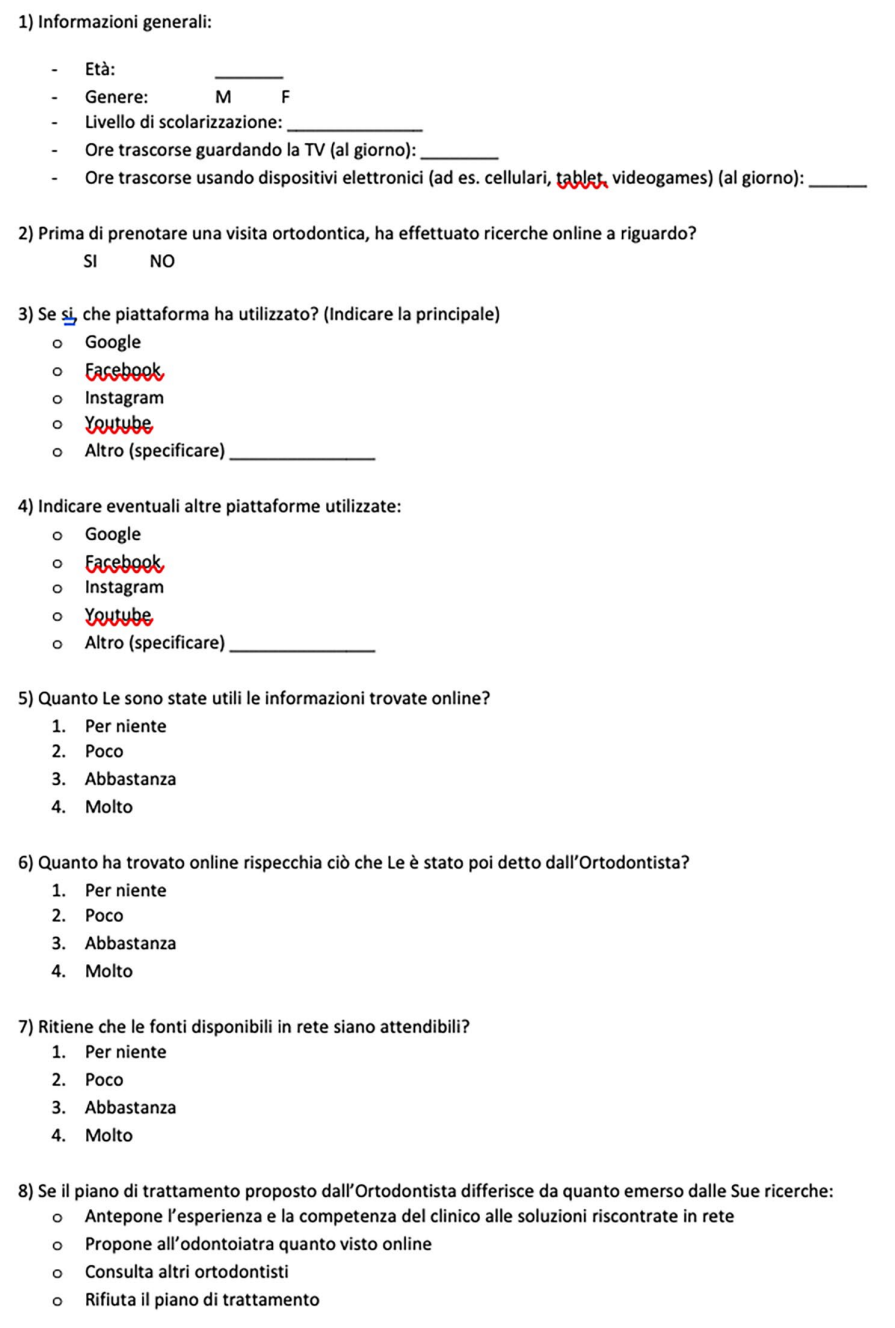
Questionnaire, first version (italian)

**Fig. 1b:**
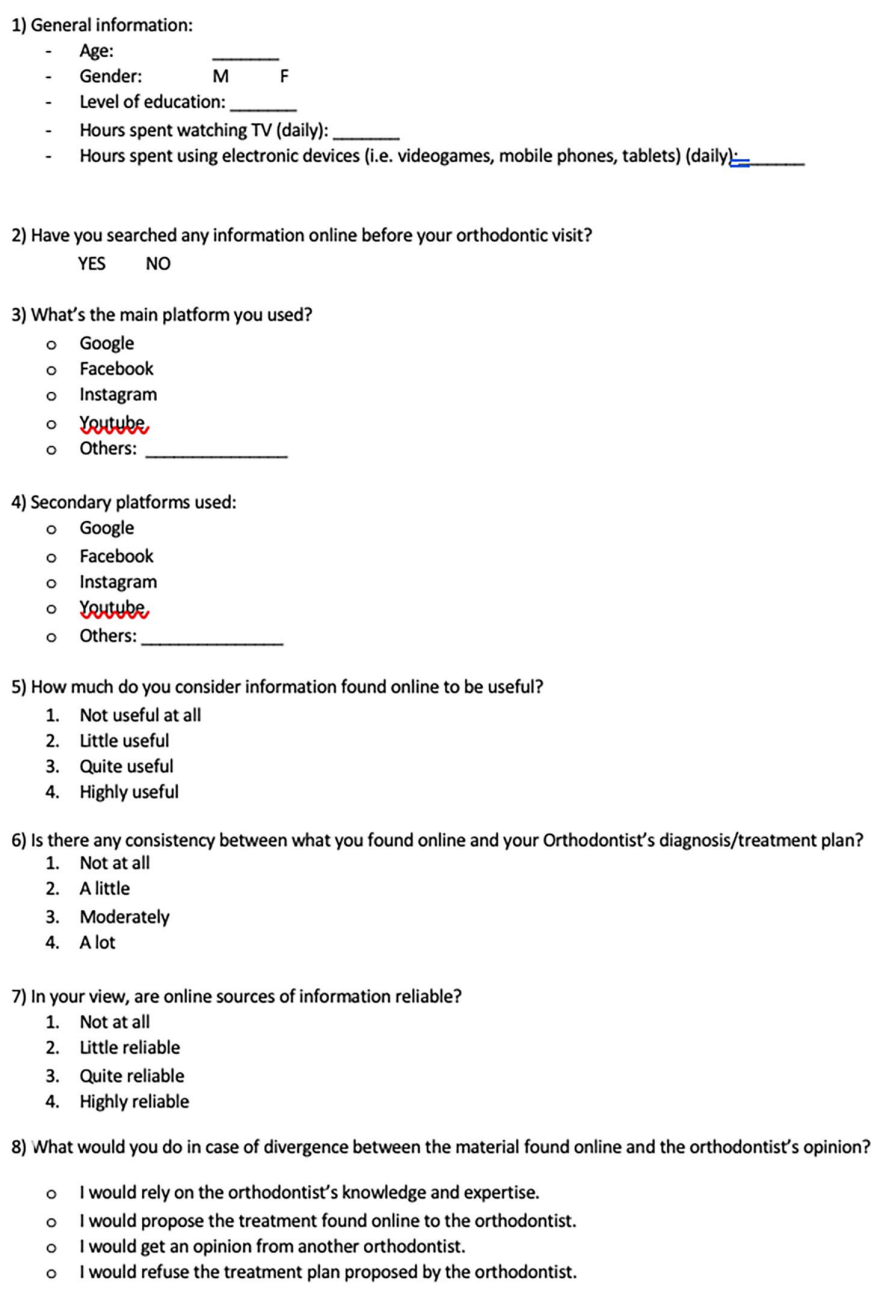
Questionnaire, first version (english)

**Fig. 2:**
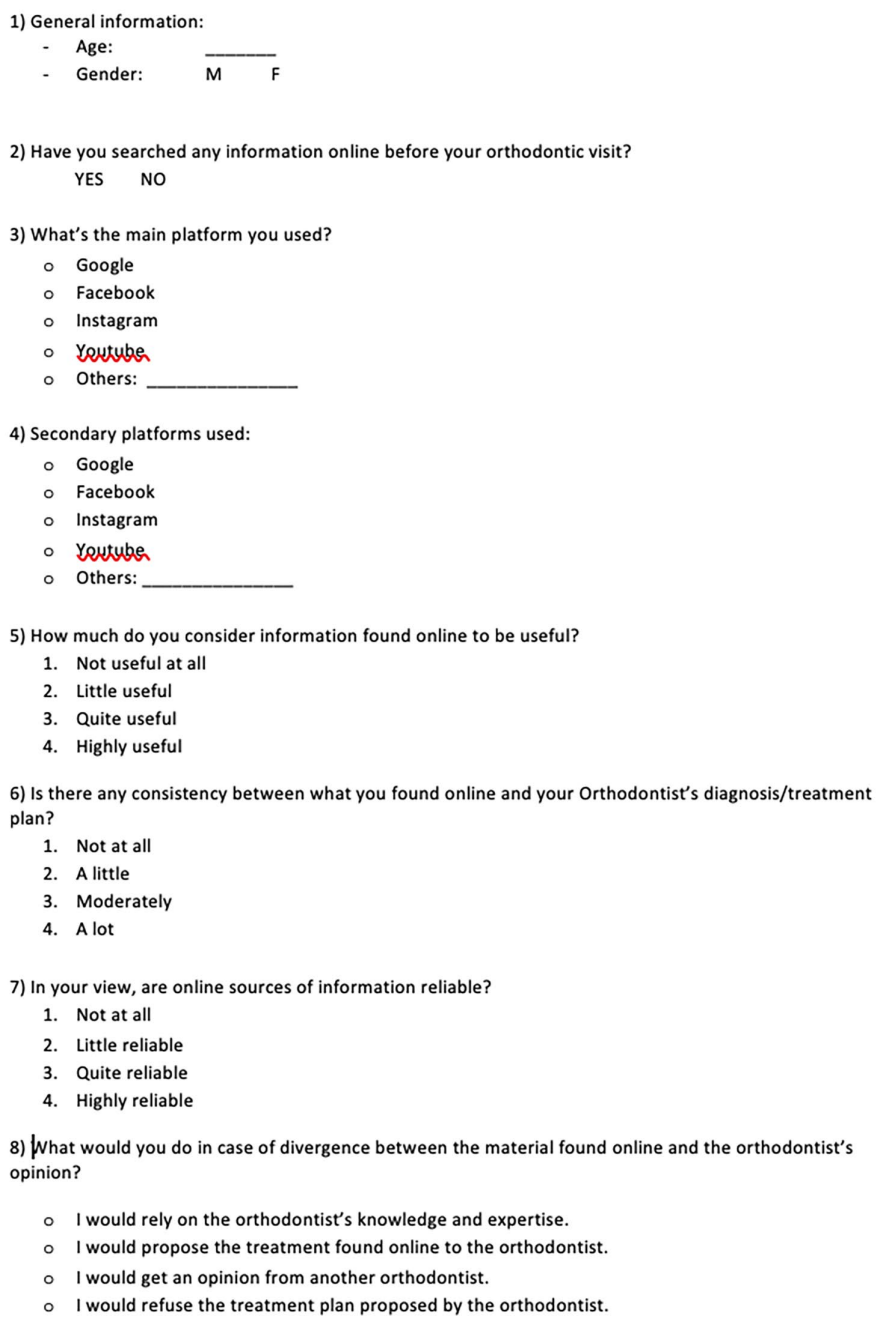
Validated Questionnaire - English version

Prior to survey deployment, a pilot study was completed. The pilot study, conducted on a small sample (N = 20), served as a validation of the survey tool including the identification and correction of any potential shortcomings. The first version of the questionnaire (Fig. 1a and b) was handed out to 20 patients, by previous research aiming to validate medical questionnaires [12, 13]. The pilot study population was composed of adult orthodontic patients and parents or legal guardians of underage orthodontic patients referring to Policlinico Universitario Agostino Gemelli (Rome); subjects ≥ 18 years old were included. Patient enrollment was carried out so that the two study groups were equally represented (DN n = 10; DI n = 10).

The initial ‘pilot’ questionnaire was designed as follows (Fig. 1):

Item one [1] of the questionnaire included open questions about the patient’s age, gender, level of education, hours spent watching TV (daily), and hours spent using electronic devices (daily).

The subsequent seven items consisted of multiple-choice questions concerning [2] online research carried out before the orthodontic visit, [3] main platform used, [4] secondary platforms used, [5] perceived usefulness of the information found online, [6] perceived consistency information found and orthodontist’s opinion, [7] perceived reliability of online sources, [8] decision-making processes in case of divergence between material found online and orthodontist’s opinion. Questions [5, 6], and [7] could be answered with a score ranging from 1 to 4, where 1 indicates “not at all” and 4 corresponds to “very much”. The remaining questions could be answered by circling “Yes” or “No” [2], and by choosing among given alternatives [3, 4, 8].

In accordance with the validation process previously carried out by Labeau et al. [14], a panel of experts in the field performed both a formal assessment of the questionnaire and an evaluation of its contents. The panel composition included 5 professionals operating at the Policlinico Universitario Agostino Gemelli (Rome) who have at least 5 years of experience as orthodontist. Formal validity consisted of a determination of question appropriateness and clarity. Content validity was determined by the collective expert rating of each question from 1 to 3, where 1 = not relevant, 2 = relevant but not necessary, and 3 = strictly necessary. Questions deemed to be unclear were reformulated. Any question rated with a mean score of 1 were removed from the questionnaire.

Aiming to create a user-friendly questionnaire, it was verified that ≥ 70% of the pilot study subjects properly understood the given questions.

As a result of the experts’ assessment of the first version of the survey, open questions about the level of education and hours spent daily either watching TV or using electronic devices were considered “not relevant” and therefore eliminated, and a new validated tool was ready to be distributed to orthodontic patients (Fig. 2). In the validated version of the questionnaire (Fig. 2), Sect. 1 “general information” was modified, through the validation process, as follows: only “age” and “gender” remained; conversely, “level of education”, “hours spent watching TV (daily)” and “Hours spent using electronic devices (i.e. videogames, mobile phones, tablets) (daily)” were removed. The other questions of the survey were not modified.

As a matter of fact, the panel of experts rated the open questions “level of education”, “Hours spent watching TV (daily)” and “Hours spent using electronic devices (daily)” a score of 1, due to the following reasons:

- “level of education”: the question was too generic, and the answers may not have been homogeneous/comparable because of the international distribution of the questionnaire;
- “hours spent watching TV (daily)”: not relevant;
- “Hours spent using electronic devices (i.e. videogames, mobile phones, tablets) (daily)”: not relevant.

The decisions were made collegially, after discussing the various points, and were approved unanimously.

During the filling of the surveys, subjects were asked if the questions were ambiguous, confounding or otherwise of dubious interpretation: all interviewees affirmed that the questions were easily intelligible and clearly stated. Afterwards, the 20 pilot surveys distributed were inspected: no question was left blank. Therefore, the questionnaire resulted to be comprehensible and ready for a wider distribution. The reliability analysis considering the items 5, 6 and 7 (Fig. 1a/b) shown a Crombach’s Alpha coefficient of 0.78.

Moreover, based on the preliminary results of the pilot study, it was possible to calculate (EpiCalc 2000) the sample size. The hypothesis assumed was: 75% digital natives and 65% digital immigrants got informed online, the confidence level was set at 95%, the power at 80%; thus, the sample size needed resulted to be equal to N = 656 (DN n = 328; DI n = 328).

The final version of the questionnaire (Fig. 2) was then distributed to participating sites.

Written informed consent was obtained for each study participant followed by instructions about the study aims protocols and methods. Participants were given the possibility to take the survey at any time during treatment, including the first visit. In order to prevent selection bias, all orthodontic patients coming for a visit from October 2020 to January 2022 were asked to participate in this study. Participation was voluntary.

Questionnaires that were not correctly and/or completely filled in, as well as questionnaires that featured unclear or conflicting answers, were excluded from the analysis.

### Statistical analysis

IBM SPSS19.0 and Excel were used for the statistical analysis of the relevant quantitative data. Descriptive analysis was performed using frequencies and percentages for qualitative variables, and mean (SD) for quantitative ones.

Univariate analysis was applied using the χ2 tests. The χ2 tests were used to analyze the relationship between the demographic characteristics of respondents and the items on the use of digital research for the decision-making process.

Items number [5, 6], and [7] were dichotomized into satisfied (answers 3 and 4) versus unsatisfied (answers 1 or 2), consistent (answers 3 and 4) versus inconsistent (answers 1 and 2), and reliable (answers 3 and 4) versus unreliable (answers 1 and 2).

α = 0.05 is used as the cut-off for significance and p < 0.05 was considered statistically significant.

## Results

The number of surveys gathered was: 478 (Italy), 286 (North Carolina), 188 (Saudi Arabia), 105 (Finland). After the exclusion of incorrectly filled questionnaires and of questionnaires that reported unclear or conflicting answers, the total of surveys was 986 (DI = 454, DN = 532).

Table 1 shows gender and type of generation of the samples studied: 68.4% was female; the digital natives were 532 (54%). The mean age of the sample was 35.6 years (SD = 13.8; min = 18; max = 72).

64.6% of the study population searched the Web for orthodontic information prior to their visit (Table 1); online research was carried out by 75.4% DN and 52% DI (Table 2).

Among those who got informed online, Google was the most popular main platform (81%), followed by YouTube (6.8%) and Instagram (4.4%) (Table 1). Google was used to a similar extent by digital natives and digital immigrants, whereas the usage of other platforms varied with age (Table 2).

In regard to the perceived usefulness of online information, similar satisfaction levels were recorded for both study groups; 68.8% digital natives and 64.1% digital immigrants considered it to be “useful” or “very useful” (Table 1).

The patients’ perception of consistency between online information and the orthodontist’s opinion didn’t significantly vary between the study groups. In fact, 51.9% digital immigrants and 44.7% digital natives observed moderate consistency; similarly, 31.3% digital immigrants and 32.5% digital natives observed little consistency (Table 2).

**Table 1:** Respondents’ demographics and answers

Variables			N	%
Countries		Italy	478	48.5
		Saudi Arabia	178	18.1
		Finland	105	10.6
		North Carolina	225	22.8
Question (1): Gender		Female	674	68.4
		Male	312	31.6
Question (1): Generation (age)		Digital immigrants	454	46
		Digital natives	532	54
Question (2): Online research prior to visit		No	349	35.4
		Yes	637	64.6
Question (3): Main platform	Google	No	121	19.0
		Yes	516	81.0
	Istagram	No	609	95.6
		Yes	28	4.4
	Youtube	No	594	93.2
		Yes	43	6.8
	Facebook	No	611	95.6
		Yes	26	4.1
	others	No	616	96.7
		Yes	21	3.3
Question (4): Secondary platform	Google	No	547	86.3
		Yes	87	13.7
	Istagram	No	594	60.2
		Yes	40	4.1
	Youtube	No	545	86.0
		Yes	89	14.0
	Facebook	No	594	93.7
		Yes	40	6.3
	others	No	596	94.0
		Yes	38	6.0
Question (5): Perceived usefulness of digital information		1	23	3.6
		2	187	29.3
		3	290	45.5
		4	138	21.6
Question (6): Consistency between digital information and orthodontist’s opinion		1	20	3.2
		2	201	32.1
		3	297	47.4
		4	109	17.4
Question (7): Perceived reliability of digital sources		1	30	4.7
		2	296	46.5
		3	246	38.6
		4	65	10.2
Question (8): Decisional process in case of discrepancy between orthodontist’s opinion and digital information		A^1^	456	71.7
		B^1^	99	15.6
		C^1^	80	12.6
		D^1^	1	0.2
Age (mean 35.58; SD 13.763; median 33.00; min 18; max 72)				

^1^ A = I would rely on the orthodontist’s knowledge and expertise; B = I would propose the treatment found online to the orthodontist; C = I would get an opinion from another orthodontist; D = I would refuse the treatment plan proposed by the orthodontist.

The perceived reliability of online sources varied significantly between the study groups (p = 0.007). In fact, while 53.8% digital immigrants considered the Web-based contents “quite reliable” (44.9%) or “highly reliable” (8.9%), almost 1 in 2 digital natives defined them as “little reliable” (Table 2). The significance of post-hoc test demonstrates a significant difference in choosing the answer 1 (“not reliable at all”) over the other alternatives, and it also proves that the percentage of answers 1 is higher in DN than in DI.

When the surveyed population was enquired about their behavioral response in a scenario in which the information found on the Web was inconsistent with the clinical judgement of the orthodontist, the majority of digital immigrants (68.8%) and digital natives (73.4%) affirmed they would trust the clinician’s expertise; 14.8% digital immigrants and 16% digital natives would propose to their dentist what they found online; digital immigrants were more likely than digital natives to either get an opinion from another orthodontist or completely refuse to be treated (Table 2).

**Table 2:** Univariate analysis by digital natives versus digital immigrants

Variables		Digital Generation				
		Digital Immigrants		Digital Natives		
		**N**	**%**	**N**	**%**	P^
Question (2): Online research prior to visit	No	218	48.0	131	24.6	**< 0.001**
	Yes	236	52.0	401	75.4	
Question (3): Main platform	Google	193	81.8	323	80.5	0.702
	Istagram	6	2.5	22	5.5	0.080^^
	Youtube	2	0.8	41	10.2	**< 0.001^^**
	Facebook	23	9.7	3	0.7	**< 0.001^^**
	others	12	5.1	9	2.3	0.055^^
Question (4): Secondary platform	Google	15	6.4	72	18.1	**< 0.001**
	Istagram	4	1.7	36	9.0	**< 0.001^^**
	Youtube	14	5.9	75	18.8	**< 0.001^^**
	Facebook	22	9.3	18	4.5	0.016^^
	others	19	8.1	19	4.8	0.093^^
Question (5): Perceived usefulness	1 + 2 (Unsatisfied)	85	35.9	125	31.2	0.223
	3 + 4 (Satisfied)	152	64.1	276	68.8	
Question (6): Consistency	1	6	2.6	14	3.6	0.227
	2	73	31.3	128	32.5	
	3	121	51.9	176	44.7	
	4	33	14.2	76	19.3	
Question (7) Perceived reliability of digital sources	1	4^a^	1.7	26^a^	6.5	**0.007**
	2	105	44.5	191	47.6	
	3	106	44.9	140	34.9	
	4	21	8.9	44	11.0	
Question (8) Decisional process	A*	163	68.8	293	73.4	0.095
	B*	35	14.8	64	16.0	
	C + D*	39	16.5	42	10.5	

* A = I would rely on the orthodontist’s knowledge and expertise; B = I would propose the treatment found online to the orthodontist; C = I would get an opinion from another orthodontist; D = I would refuse the treatment plan proposed by the orthodontist;

bold: p < 0.05.

^ : p-value of Pearson Chi Square test

^^: p-value of Fisher Exact test

a: indicates statistical significance at the adjusted α level of 0.00625 applying the post-hoc test of chi-square test.

Analyzing the study population based on their decisional processes in the event of discrepancy between the orthodontist’s opinion and Internet-based contents (Table 3), the following observations were made:

- 78.6% of the surveyed men would trust their orthodontist, whereas approximately 1/3 of the surveyed women would either propose the treatment found online, get a second opinion or refuse the treatment option given by their orthodontist. The differences observed between genders are statistically significant (p < 0.05), but considering the post-hoc analysis it was not confirmed;
- A significant correlation was observed between decisional processes and the level of satisfaction reached with patient-driven digital research (p = 0.002). The post Hoc analysis highlighted that the unsatisfied group had a significant high percentage in answering A (“I would rely on the orthodontist’s knowledge and expertise”) and B (“I would propose the treatment found online to the orthodontist”). 80% of patients who were not satisfied with their Internet searches relied on the clinician’s judgement, whereas, among those who believed online information to be “quite useful” or “highly useful”, 18% would propose treatment options that emerged from the digital search to the orthodontist. 14% would either consult another orthodontist or refuse to be treated.
- The perceived consistency between digital information and the orthodontists’ opinion did not show any influence on patients’ decisional processes (p = 0.209);
- The perceived reliability of digital sources greatly impacted patients’ decisional processes (p < 0.001). 78.8% of those who judged online sources as “not reliable at all” or “little reliable” chose to rely on the orthodontists expertise; conversely, among those who judged online sources as “quite reliable” or “highly reliable”, more than 1 in 3 would either propose the treatment found online, consult another doctor or refuse the cures.

**Table 3:** Univariate analysis by decisional processes

Variables		Question (8) Decisional process in case of discrepancy between orthodontist’s opinion and digital information							
		A*		B*		C*+D*			
		N	% row % column	N	% row % column	N	% row % column		P^
Question (1) Gender	Male	147	78.6	20	10.7	20	10.7	100.0%	**0.036**
			32.2		20.2		24.7		
	Female	309	68.8	79	17.6	61	13.6	100.0%	
			67.8		79.8		75.3		
Country	Italy	219	68.2	64	19.9	38	11.8	100.0%	Linear-by-Linear Association 0.447
			48.0		64.6		46.9		
	Saudi Arabia	114	78.6	15	10.3	16	11.0	100.0%	
			25.0		15.2		19.8		
	Finland	36	100.0	0	0	0	0	100.0%	
			7.9		0		0		
	North Carolina	87	64.9	20	14.9	27	20.1	100.0%	
			19.1		20.2		33.3		
Question (2)	Google	375	73.0	77	15.0	62	12.1	100.0%	0.393
			82.2		78.6		76.5		
	Other	81	66.9	21	17.4	19	15.7	100.0%	
			17.8		21.4		23.5		
Question (5): Perceived usefulness	Unsatisfied	169^a^	80.5	20 ^a^	9.5	21	10.0	100.0%	**0.002**
			37.1		20.4		25.9		
	Satisfied	287^a^	67.5	78 ^a^	18.4	60	14.1	100.0%	
			62.9		79.6		74.1		
Question (6): Consistency	Inconsistent	163	74.4	35	16.0	21	9.6	100.0%	0.209
			36.5		35.7		26.3		
	Consistent	284	70.0	63	15.5	59	14.5	100.0%	
			63.5		64.3		73.8		
Question (7) Perceived reliability of digital sources	Unreliable	257^a^	78.8	35 ^a^	10.7	34	10.4	100.0%	**< 0.001**
			56.4		35.7		42.0		
	Reliable	199^a^	64.4	63 ^a^	20.4	47	15.2	100.0%	
			43.6		64.3		58.0		

* A = I would rely on the orthodontist’s knowledge and expertise; B = I would propose the treatment found online to the orthodontist; C = I would get an opinion from another orthodontist; D = I would refuse the treatment plan proposed by the orthodontist;

bold: p < 0.05.

P^: p-value of Pearson Chi Square

a: indicates statistical significance at the adjusted α level of 0.0083 applying the post-hoc test of chi-square test

## Discussion

In this study, we assessed orthodontic patients’ attitudes toward Web-based information as well as the psychometric implications of orthodontic-themed online research. The behavioral effect of social media on orthodontic patients had previously been investigated in the USA by Nelson and Shroff [3], who observed that social media can be used by orthodontists not only as a communications media but also as a marketing tool. It’s noticeable in their work, as well as in ours, that the use of online platforms decreases with age.

Henzell et al. [15] performed a qualitative analysis of orthodontic-related posts on Twitter in New Zealand. They observed that adolescents’ posts concerned experiences and feelings about orthodontic treatment, and only a small number of informational posts were found. These results differ from ours - although such differences may reflect geographical differences, and it may also depend on the very selective choice of online platform made by said Authors. In fact, Twitter may not be the platform of choice for informational content; in our observations, Google resulted to be the most used digital platform by far, and even amongst those who didn’t use it as their principal source it is often chosen as a secondary one (Table 1). YouTube, Instagram and Facebook were used by 15.3% of the respondents, whereas platforms other than the above-mentioned ones barely reached 3.3% (Table 1). Moreover, our results show that the preferred digital platform significantly varies with age (p < 0.001). In fact, YouTube is used as main source of orthodontic information mostly by digital natives, conversely, Facebook is preferred mainly by digital immigrants (Table 2).

It is also noticeable from our investigation that digital natives are more likely to use the Internet to acquire orthodontic information (p < 0.001), and that not only fewer digital immigrants searched the Web, but also their digital scouting was less wide-ranging. Remarkably, secondary platforms were selected by more than a half of the digital natives, whereas only 31.4% digital immigrants declared to have used a secondary platform (Table 2).

To our knowledge, this is the first study to assess the psychometric implications of orthodontic-themed Web-searches on patients’ decisional processes.

In our experience, female patients incorporated information from Web searches in their decisions more than males, who relied more on the orthodontist’s advice (Table 3).

Furthermore, the likelihood to raise concerns about the clinician’s judgement appears to be related to the perceived usefulness of online sources. In fact, patients who were satisfied with the information available online were also more reluctant to cast it aside in case it differed from the orthodontist’s opinion, and 1 in 3 would not rely solely on the dentist’s judgment. On the other hand, more than 80% of the patients who were unsatisfied with their searches preferred to trust the orthodontist (Table 3).

The perceived reliability of digital sources showed an even more significant influence on patients’ behaviour (p < 0.001). Indeed, patients who judged online sources to be reliable were more likely to hold on to the information found on the Web, and 35.6% would propose what they found, consult other dentists or refuse to be treated (Table 3). Furthermore, not only digital immigrants were more likely than digital natives to perceive online sources of information as reliable, but they were also more reluctant to put aside the internet-based information when it differed from the orthodontist’s opinion, as 16.5% would either get an opinion from another orthodontist or completely refuse to be treated (Table 2).

The trends described above should be taken into account during patient’s interview in order to build a more solid doctor-patient relationship. As a matter of fact, it is advisable to ask patients about their searches on the Web, discuss the results together and give them correct and exhaustive explanation of the topic. Eventually, the orthodontist should recommend evidence-based and reliable websites for further researches; this way, patients could acquire valid, evidence-based knowledge on their clinical condition without the risk to fall for inexact, misleading and non-scientific information - which is abundant and easy to run into on the Web [16]. In particular, orthodontists and general dentists should educate their patients to rely on Websites in which sources are clearly stated, and to prefer contents written by professionals or scientific societies over blogs, reports of personal experiences and other types of digital material produced by laypersons. Additionally, patients who wish to dig further into orthodontic topics could be provided with links to the websites of national orthodontic scientific associations, such as British Orthodontic Society (BSO) (UK) [17], Società Italiana di Ortodonzia (SIDO) (Italy) [18, 19] and American Association of Orthodontists (AAO) (USA) [20], which feature informative contents specific for patients.

It is particularly important to build a well-founded doctor-patient relationship not only for ethical reasons and for the patient’s wellbeing, but also because the tendency to trust the orthodontist didn’t show any correlation with the perceived consistency between online information online and dentist’s opinion, whereas the satisfaction with online contents impacted very significantly on the patients’ decisional processes (Table 3). This implies that the likelihood of acceptance of the proposed treatment plan is affected by the patient’s satisfaction with their online searches but not by the found contents per se.

It is important to highlight the limitations of our investigation. First, due to the COVID-19 pandemic, the survey distribution varied among centers participating in the study. While statistical significance was reached, it was not possible to draw conclusions regarding potential differences among Countries. Second, the survey was distributed in both public and private structures, but patients referring to the former and to the latter were not compared; additional studies must be conducted to assess these parameters. Finally, the pediatric patient is not investigated in this study.

## Conclusions

Our investigation highlighted that orthodontists should be aware that the majority of patients use the Internet as a source for orthodontic information, and that Internet use differs among generations. When discussing treatment plan options, men are more likely to rely on the orthodontist’s judgement than women, and patients who perceive the information found on the Web as either useful or reliable don’t easily discard it, even if it is inconsistent with the orthodontist’s opinion.

## Data Availability

The datasets used and/or analysed during the current study are available from the corresponding author on reasonable request.

## List of abbreviations

DN: digital natives
DI: digital immigrants
